# Perceptions of neighbourhood quality, social and civic participation and the self rated health of British adults with intellectual disability: cross sectional study

**DOI:** 10.1186/1471-2458-14-1252

**Published:** 2014-12-09

**Authors:** Eric Emerson, Chris Hatton, Janet Robertson, Susannah Baines

**Affiliations:** Centre for Disability Research, Lancaster University, Lancaster, LA1 4YT UK; Centre for Disability Research and Policy, University of Sydney, Sydney, Australia

**Keywords:** Health, Social participation, Civic participation, Neighborhoods

## Abstract

**Background:**

There is extensive evidence from research undertaken on general population samples that people who have more extensive and closer social networks and people who report feeling connected to their local community tend to have better health. However, relatively few studies have examined the relationship between the social connectedness of people with intellectual disabilities and their health.

**Methods:**

Secondary analysis of data from *Understanding Society,* a new longitudinal study focusing on the life experiences of UK citizens. We identified 279 participants aged 16–49 (1.1% of the unweighted age-restricted sample) as having intellectual disability, and 22,927 as not having intellectual disability. Multivariate logistic regression was used to investigate between group differences adjusting for potential confounding personal characteristics (e.g., gender).

**Results:**

British adults with intellectual disability had less favorable perceptions of important neighborhood characteristics and lower levels of social and civic participation than their non-disabled peers. Favorable perceptions of important neighborhood characteristics and higher levels of social and civic participation were associated with more positive self-rated health for adults with and without intellectual disability. For adults with intellectual disability this was particularly the case with regard to employment, feeling safe outside in the dark and being able to access services when needed. The between-group differences in perceptions of important neighborhood characteristics and levels of social and civic participation accounted for a significant proportion of the elevated risk for poorer self-rated health observed among adults with intellectual disability.

**Conclusions:**

This study provides evidence to suggest that the health inequalities experienced by people with intellectual disabilities may be partially attributable to their less favorable perceptions of important neighborhood characteristics and lower levels of social and civic participation.

## Background

Intellectual disability refers to a significant general impairment in intellectual functioning that is acquired during childhood, typically operationalised as scoring more than two standard deviations below the population mean on a test of general intelligence [1, 2]. While estimates of the prevalence of intellectual disability vary widely, it has been estimated that approximately 2% of the adult population have intellectual disability [3, 4]. People with intellectual disability have significantly higher age adjusted rates of mortality and morbidity than their non-disabled peers [1, 5–8]. This evidence, when combined with exposés of failings in healthcare systems [6, 9–11] and increased attention to the human rights of disabled people [12], has led regulatory bodies and governments to stress the importance of reducing the health inequalities experienced by people with intellectual disability [13–17].

Recent research has drawn attention to the role that increased rates of exposure to common social determinants of health (especially indicators of low socio-economic position) may play in accounting for the poorer health of people with intellectual disabilities [1, 18–20]. However, few studies have examined the relationship between indicators of either neighborhood quality or the social connectedness of people with intellectual disabilities (e.g., levels of civic engagement) and their health [21–23]. This may be an important omission given that: (1) there is extensive evidence from general population studies that people who have more extensive and closer social networks, people who report feeling connected to their local community and people living in more supportive neighborhoods tend to have better health [24–34]; and (2) there is also extensive evidence that people with intellectual disabilities often have highly restricted social networks and live in less supportive neighborhoods [23, 35–44].

The sparse literature on the association between the social connectedness of people with intellectual disabilities and their health has reported positive associations between better health and higher frequency of contact with friends with intellectual disability [21–23], being in paid employment [22, 23] and higher frequency of participation in community activities [21, 22].

The primary exposures of interest are perceived neighborhood quality, social and civic participation. The primary outcome of interest in this study is the self-rated health of British adults with intellectual disability. The specific aims of the study were: (1) to describe levels of exposure to perceived neighborhood quality, social and civic participation among British adults with and without intellectual disability; (2) to estimate the strength (and statistical significance) of the relationship between perceived neighborhood quality, social and civic participation and self-rated health among British adults with and without intellectual disability; and (3) to estimate the strength (and statistical significance) of the relationship between intellectual disability and self-rated health prior to and following adjusting for any potential confounding effects due to between group differences in exposure to socio-economic disadvantage, perceived neighborhood quality and civic participation.

## Methods

The present study involved secondary analysis of data collected in *Understanding Society*, a new longitudinal study focusing on the life experiences of UK citizens. Data were downloaded from the UK Data Archive (http://www.data-archive.ac.uk/). Full details of the surveys’ development and methodology are available in a series of reports [45–52], key aspects of which are summarized below.

### Samples

In the first wave of data collection (undertaken between January 2009 and December 2011), random sampling from the Postcode Address File in Great Britain and the Land and Property Services Agency list of domestic properties in Northern Ireland identified 55,684 eligible households. Interviews were completed with 50,994 individuals aged 16 or older from 30,117 households, giving a household response rate of 54% and an individual response rate within co-operating households of 86% [45, 52]. At Wave 3 interviews were completed with 49,768 individuals aged 16 or older from 27,715 households, giving an individual response rate within co-operating households of 90% [52]. The follow-up response rate from Wave 2 to Wave 3 was 81% [52].

### Procedures

Data collection for all variables used in the present paper was undertaken using Computer Assisted Personal Interviewing.

### Measures

#### Intellectual disability

*Understanding Society* does not include information on the formal diagnosis of intellectual disability. As a result, we identified adults with intellectual disability on the basis of the results of cognitive testing undertaken at Wave 3 and self-reported educational attainment. The vast majority of children with intellectual disability have very low educational attainment [53]. As a result, low self-reported educational attainment (no educational qualifications) was used as a selection criterion as evidence that low cognitive ability may have originated in childhood (one of the defining characteristics of intellectual disability). Due to historical changes in educational qualifications and attainment in the UK, we restricted our analysis to the age range 16–49.

In Wave 3 a battery of five cognitive tests was used to assess memory (two tests) and cognitive functioning (three tests; Number Series, Verbal Fluency, Numerical Ability) [54]. The Number Series test was developed for use in the US Health and Retirement Study (HRS) [55]. The Verbal Fluency test has been used in the English Longitudinal Study of Ageing (ELSA) [56], the German Socio-economic Panel Study [57] and the National Survey of Health and Development [58]. The Numerical Ability test was taken from ELSA and some portions of it have been used in the HRS and Survey of Health, Ageing and Retirement in Europe [59].

First, we standardized test scores on the latter three tests to have a mean of zero and standard deviation of one. Second, we used linear regression to impute missing standardized test scores from obtained scores on completed tests. No other variables were used in the imputation process. This led to the imputation of Numeric Ability scores for 153 participants (0.6% of the used sample), Verbal Fluency scores for 141 participants (0.6%) and Number Series scores for 1214 participants (4.9%). Third, we used principal components analysis to extract the first component (which accounted for 63% of the variance) from the three scales as an estimate of general intelligence [60]. Fourth, we identified participants as having intellectual disability if they scored lower than two standard deviations below the mean on the extracted component (the conventional cut-off point for defining intellectual disability used in ICD-10) and had no educational qualifications. This identified 294 participants (1.2% of the unweighted age-restricted sample) as having intellectual disability. An additional 532 participants scored less than two standard deviations below the mean on the extracted component but did have educational qualifications.

Fifth, we included in the intellectual disability group five participants who gave consent for testing but for whom all three tests were terminated due to their inability to understand the test instructions, and also had no educational qualifications. The complete procedure identified 299 participants (1.2% of the unweighted age-restricted sample) as having intellectual disability.

#### Health

Self-rated health was evaluated by a single question incorporating five possible response options: ‘*In general, would you say your health is … (1) excellent, (2) very good, (3) good, (4) fair, (5) poor*’. Data were recoded into a binary variable; excellent/very good/good versus fair/poor.

#### Perceptions of neighborhood quality

We extracted data from eight questions relating to perceptions of neighborhood quality.

1. ‘*Overall, do you like living in this neighbourhood (Yes/No)?*’
2. ‘*Are you able to access all services such as healthcare, food shops or learning facilities when you need to (Yes/No)?*’
3. ‘*I am going to read out a set of statements that could be true about your neighbourhood. Please tell me how much you agree or disagree that each statement describes your neighbourhood (1 Strongly agree, 2 Agree, 3 Neither agree nor disagree, 4 Disagree, 5 Strongly disagree): (a) First, this is a close-knit neighbourhood; (b) People around here are willing to help their neighbours; (c) People in this neighbourhood can be trusted; (d) People in this neighbourhood generally don't get along with each other.*’ Data were recoded into binary variables; 1–3 v 4–5 for positively worded questions (a-c), 1–2 v 3–5 for question (d).
4. ‘*Now I have some questions about crime. Do you ever worry about the possibility that you, or anyone else who lives with you, might be the victim of crime? Is this a big worry, a bit of a worry, or an occasional doubt?*’ Data were recoded into a binary variable; crime is a big worry v not.
5. ‘*How safe do you feel walking alone in this area after dark? (1 Very safe, 2 Fairly safe, 3 A bit unsafe, 4 Very unsafe, 5 SPONTANEOUS: Never goes out after dark)*’. Data were recoded into a binary variable fairly safe/very safe v not.

Exploratory analysis of the resulting data indicated that the recoded binary variables from Q1 and Q3 (a-d) showed acceptable internal consistency (alpha = 0.69). As a result, they were combined into a five item scale of ‘neighborhood quality’ (range 0–5 with 5 being highest quality) that was then recoded due to the small proportions of people scoring 0–2 into a three item scale (0–3, 4, 5).

#### Civic & social participation

We extracted data from five questions relating to civic and social participation.

1. ‘*How many close friends would you say you have?*’ Data were recoded into a binary variable; two or more close friends v not.
2. ‘*Do you go out socially or visit friends when you feel like it (Yes/No)?*’
3. ‘*What stops you from going out socially or visiting friends when you want to (1 Too busy/not enough time, 2 Financial reasons, 3 A health condition, illness or impairment, or disability, 4 No public transport available, 5 Public transport is infrequent or unreliable, 6 Can't access the public transport that is available, 7 No access to a car as a driver or passenger, 8 Nowhere to go in the area, 9 No-one to go with, 10 Attitudes of other people, 11 Fear of crowds, 12 Fear of crime, 13 Anxiety/lack of confidence, 14 Caring responsibilities, 97 Other reasons)?*’
4. ‘*Please tell me how easy or difficult you would find it to visit family or relatives when you need to (1 Very difficult, 2 Difficult, 3 Neither difficult nor easy, 4 Easy, 5 Very easy, 6 Has no family).*’ Data were recoded into a binary variable; Easy/very easy v not.
5. ‘*Are you currently a member of any of the kinds of organisations on this card (1 Political party, 2 Trade Unions, 3 Environmental group, 4 Parents'/School Association, 5 Tenants'/Residents' Group or Neighbourhood Watch, 6 Religious group or church organisation, 7 Voluntary services group, 8 Pensioners group/organisation, 9 Scouts/Guides organisation, 10 Professional organisation, 11 Other community or civic group, 12 Social Club/Working men's club, 13 Sports Club, 14 Women's Institute/Townswomen's Guild, 15 Women's Group/Feminist Organisation, 16 Other group or organisation, 96 SPONTANEOUS None of these)*’. Data were recoded into a binary variable; member of one or more organization vs not.

#### Socio-economic disadvantage

Self-assessed financial status was assessed at Wave 3 by a single item: ‘How well would you say you yourself are managing financially these days? Would you say you are… 1 Living comfortably, 2 Doing alright, 3 Just about getting by, 4 Finding it quite difficult or 5 finding it very difficult?’ Data were recoded into a binary variable; living comfortably/doing alright v not.

### Approach to analysis

Our approach to analysis was undertaken in five stages. First, we made simple bivariate comparisons between participants with and without intellectual disability with regard to available socio-demographic characteristics that may have a potential association with health (e.g., financial strain, gender).

Second, we made adjusted bivariate comparisons (using multivariate binary logistic regression) between participants with and without intellectual disability with regard to exposure to perceived neighborhood characteristics and reported levels of social/civic participation. These comparisons were adjusted to take account of any potential confounding effects of the socio-demographic characteristics investigated in Stage 1 that were or closely approached being statistically significant different between the two groups.

Third, we estimated the strength of the association between measures of exposure to perceived neighborhood characteristics and reported levels of social/civic participation and the primary outcome of interest (self-rated health) separately for participants with and without intellectual disability, adjusting for the same socio-demographic characteristics as in Stage 2.

Fourth, we employed binary logistic regression to estimate the unique association between indicators of socio-demographic characteristics of participants, perceived neighborhood characteristics, reported levels of social/civic participation and the primary outcome of interest (self-rated health) for participants with intellectual disability.

Finally, we used multivariate logistic regression to estimate the extent to which the poorer self-rated health of participants with intellectual disability could potentially be attributed to confounding between group differences in: (1) demographics; (2) socio-economic advantage; and (3) differences in perceived neighborhood quality, social and civic participation.

### Ethical approval

Understanding Society is designed and conducted in accordance with the ESRC Research Ethics Framework and the ISER Code of Ethics. The University of Essex Ethics Committee approved Waves 1–5 of Understanding Society. Approval from the National Research Ethics Service was obtained for the collection of biosocial data by trained nurses in Waves 2 and 3 of the main survey (Understanding Society – UK Household Longitudinal Study: A Biosocial Component, Oxfordshire A REC, Reference: 10/H0604/2).

## Results

In the first stage of analysis we made simple bivariate comparisons between participants with and without intellectual disability with regard to available demographic characteristics that have a potential association with health (Table 1). As can be seen, participants with intellectual disability were significantly more likely than other participants to be older, to have children and to be more likely to experience socio-economic disadvantage. There was also a non-significant trend for them to be women. As a result, all subsequent estimates of effect sizes are adjusted to take account of between-group differences in age, gender, having children and socio-economic disadvantage.

**Table 1:** **Selected socio-demographic characteristics of participants**

Variable	Intellectual disability (n = 279)	No intellectual disability (n = 22,927)	OR/p
Women	62%	57%	1.26 (0.99-1.59)
Age 30-49	74%	65%	1.58** (1.22-2.05)
De facto married or separated/widowed	63%	66%	0.89 (0.70-1.13)
Has children	39%	32%	1.33* (1.05-1.68)
‘Doing all right’ or ‘living comfortably’	37%	57%	0.43*** (0.34-0.55)

OR = Odds Ratio.

*p < 0.05, **p < 0.01, ***p < 0.001.

In the second stage of analysis we made adjusted bivariate comparisons (using multivariate binary logistic regression) between participants with and without intellectual disability with regard to the dependent variables perceived neighborhood characteristics and reported levels of social/civic participation (Table 2). As can be seen, participants with intellectual disability were significantly less likely than other participants to report positive neighborhood characteristics and social/civic participation once results were adjusted to take account of between-group differences in age, de facto marital status and socio-economic disadvantage. Most of the effect sizes were of moderate magnitude (OR <0.54 or >1.88), with having two or more close friends, being a member of a civic organization and being employed for 16 or more hours per week being large effect sizes (OR <0.33 or >3.00) [61].

**Table 2:** **Perceptions of neighborhood quality, social and civic participation of British adults with and without intellectual disability**

Variable	Intellectual disability (n = 299)	No intellectual disability (n = 22,927)	OR/p
Neighborhood			
Neighborhood quality: High	53%	65%	0.57*** (0.42-0.75)
Medium	23%	20%	0.78 (0.55-1.09)
Low	24%	16%	1.0 (reference)
Crime not a big worry	87%	94%	0.68* (0.47-0.98)
Feels safe outside in dark	59%	78%	0.44*** (0.35-0.57)
Can access local services when needed	96%	98%	0.58 (0.32-1.03)
Civic & Social Participation			
Member of civic organization	16%	49%	0.20*** (0.15-0.28)
Employed 16+ hours per week	15%	58%	0.13*** (0.09-0.17)
Easy to visit family	52%	68%	0.55** (0.44-0.70)
Two or more close friends	67%	92%	0.20*** (0.15-0.25)
Goes out socially	74%	88%	0.47*** (0.36-0.62)

OR = Odds Ratio.

*p < 0.05, **p < 0.01, ***p < 0.001.

OR estimates adjusted to take account of between-group differences in gender, age, socio-economic disadvantage and having children.

For participants with intellectual disability, the five most common reasons for not going out socially were: a health condition, illness, impairment or disability (36%); financial (20%); nobody to go out with (16%); too busy (16%); and caring responsibilities (11%). For participants without intellectual disability, the five most common reasons for not going out socially were: too busy (47%); caring responsibilities (30%); financial (28%); a health condition, illness, impairment or/disability (10%); and nobody to go out with (8%).

In the third stage of analysis we estimated the strength of the association between measures of perceived neighborhood characteristics and reported levels of social/civic participation and self-rated health separately for participants with and without intellectual disability (binary logistic regression adjusted to take account of gender, age, having children and socio-economic disadvantage) (Table 3). As can be seen, with one exception (membership of community organisation for participants with intellectual disability) more positive perceived neighborhood characteristics and higher reported levels of social/civic participation were associated with more positive self-rated health for participants with and without intellectual disability. While for participants without intellectual disability all these comparisons were highly statistically significant, for participants with intellectual disability only six of the nine comparisons reached the conventional level of statistical significance. However, four of these associations (crime not being a big worry, going out socially, neighborhood quality and feeling safe outside in the dark) were of moderate effect size, and two (ability to access local services when needed, being employed for 16 or more hours per week) were large [61].

**Table 3:** **Estimated strength of association (odds ratios) between indicators of perceptions of neighborhood quality, social and civic participation and the positive self-rated health of british adults with and without intellectual disability**

Variable	Intellectual disability (n = 299)	No intellectual disability (n = 22,927)
Neighborhood		
Neighborhood quality: High	2.02* (1.07-3.81)	1.99*** (1.81-2.18)
Medium	1.99 (0.94-4.22)	1.67*** (1.49-1.88)
Low (reference)	1.0	1.0
Crime not a big worry	2.22* (1.03-4.78)	2.31*** (2.06-2.60)
Feels safe outside in dark	2.15** (1.27-3.64)	1.90*** (1.75-2.07)
Can access local services when needed	4.45* (1.22-16.21)	2.10*** (1.72-2.57)
Civic & Social Participation		
Member of civic organization	0.87 (0.43-1.76)	1.69*** (1.56-1.83)
Employed 16+ hours per week	4.92** (1.88-12.83)	2.10*** (1.94-2.27)
Easy to visit family	1.36 (0.81-2.28)	1.31*** (1.22-1.42)
Two or more close friends	1.36 (0.79-2.34)	1.90*** (1.70-2.12)
Goes out socially	1.88* (1.05-3.37)	2.01*** (1.83-2.21)

*p < 0.05, **p < 0.01, ***p < 0.001.

Estimates adjusted to take account of effects of gender, age, having children and socio-economic disadvantage.

In the fourth stage of the analysis we employed binary logistic regression to estimate the unique association between indicators of socio-demographic characteristics of participants with intellectual disability, perceived neighborhood characteristics, reported levels of social/civic participation and the positive self-rated health (Table 4). Variables were entered in two blocks: (1) age, gender, whether participants had children and socio-economic disadvantage; (2) perceived neighborhood characteristics and reported levels of social/civic participation. In order to reduce the ratio of variables to participants, only measures that showed significant adjusted associations with self-rated health (Table 3) were entered into the model in a forward stepwise conditional procedure with criteria or variable entry being p < 0.1. As can be seen, more positive self-rated health was statistically uniquely associated with younger age, socio-economic advantage, being employed for 16 or more hours per week and feeling safe outside in the dark. However, while not statistically significant the unique association between being able to access services and positive self-rated health represented a large effect size [61]. The robustness of the model was examined by forcing entry of the non-included variables individually and in combinations, none of which changed the overall results.

**Table 4:** **Estimated strength of unique association (odds ratios) between indicators of perceptions of neighborhood quality, social and civic participation and the positive self-rated health of British adults with intellectual disability**

Variable	OR/p
Female gender	1.27 (0.63-2.57)
Age 30+	0.29** (0.14-0.60)
Has children	1.29 (0.65-2.53)
Socio-economic advantage	2.65** (1.44-4.88)
Feels safe outside in dark	1.90* (1.11-3.27)
Can access services when needed	3.30 (0.83-13.07)
Employed 16+ hours per week	4.31** (1.64-11.31)

OR = Odds Ratio.

*p < 0.05, **p < 0.01.

Finally, given the similarity in the associations between these indicators of perceived neighborhood quality, social and civic participation and self-rated health among participants with and without intellectual disability, we used multivariate logistic regression to estimate the extent to which the poorer self-rated health of participants with intellectual disability (OR = 0.26, 0.19-0.34, p < 0.001) could potentially be attributed to between group differences in: (1) demographics; (2) socio-economic advantage; and (3) differences in perceived neighborhood quality, social and civic participation. Adjusting for between group differences in demographics slightly reduced estimated risk (OR = 0.28, 0.21-0.37). Adjusting for between group differences in demographics and socio-economic advantage reduced the estimated risk further (OR = 0.31, 0.23-0.41). Adjusting for between group differences in demographics, socio-economic advantage and differences in perceived neighborhood quality, social and civic participation significantly reduced the estimated risk further (OR = 0.50, 0.36-0.69).

## Discussion

Our results indicate that: (1) British adults with intellectual disability have less favorable perceptions of important neighborhood characteristics and lower levels of social and civic participation than their non-disabled peers; (2) favorable perceptions of important neighborhood characteristics and higher levels of social and civic participation are associated with more positive self-rated health for adults with and without intellectual disability; (3) for adults with intellectual disability this is particularly the case with regard to employment and social contact with friends; (4) the between-group differences in perceptions of important neighborhood characteristics and levels of social and civic participation may account for a significant proportion of the elevated risk for poorer self-rated health observed among adults with intellectual disability.

These results add to existing knowledge about the health inequalities faced by people with intellectual disability in four important ways. First, they are based on the analysis of contemporary population-based sampling frames, a relative rarity in this field of study [1].

Second, being based on samples drawn from general households, participants are likely to include adults with less severe intellectual disability who may not be in receipt of specialized disability services. Given that most intellectual disability research is based on convenience samples drawn from the users of specialized disability services (typically people with more severe intellectual disability), very little is currently known about the health or well-being of the group that has been termed the ‘hidden majority’ of adults with (mild) intellectual disability [62–64].

Third, the results contribute to the very limited literature on the relationship between perceptions of important neighborhood characteristics, levels of social and civic participation and the health of people with intellectual disabilities. The results are consistent with previous studies in highlighting the potential importance of contact with friends and paid employment to the health of adults with intellectual disability [21–23]. Finally, this is the first study (of which we are aware) which provides evidence to suggest that the health inequalities experienced by people with intellectual disabilities may be partially attributable to their less favorable perceptions of important neighborhood characteristics and lower levels of social and civic participation, in addition to their increased risk of exposure to low socio-economic position.

However, there are six limitations to the study that should be kept in mind when considering the salience and implications of these results. First, while intellectual disability was identified on the basis of tests of cognitive ability, we have only indirect evidence (through reported lack of educational attainment) that their cognitive impairments may have originated in childhood. Second, the use of a general household sampling frame excludes people with (primarily more severe) intellectual disability living in institutional forms of residential care. Third, the consent and interview procedures used in *Understanding Society* are also likely to exclude people with more severe intellectual disability from participating. Consequently, the results are likely to be particularly relevant to understand the health of British adults with less severe intellectual disability. Fourth, the sole reliance on self-report measures introduces the possibility that some of the observed associations may reflect general evaluative biases of participants. Fifth, no reasonable adjustments were made to the interview process to take account of possible intellectual impairments among participants. As a result, some participants with intellectual disability may have found some questions confusing, reducing the validity of their responses.

Finally, while the cross-sectional analyses presented in this paper are consistent with the hypothesis that the poorer health of adults with intellectual disability may be partially attributable to their living conditions (in this case less favorable perceptions of important neighborhood characteristics and lower levels of social and civic participation), the cross-sectional nature of the data do not allow us to rule out other explanations (e.g., people with intellectual disability are more susceptible to social exclusion and downward social mobility if they have poor health than their non-disabled peers).

## Conclusions

Recent research has drawn attention to the role that increased rates of exposure to common social determinants of health (especially indicators of low socio-economic position) may play in accounting for the poorer health of people with intellectual disabilities. Our results add to the very sparse literature which is consistent with the hypothesis that the higher rates of social exclusion experienced by people with intellectual disabilities may also partially account for their relatively poorer health status. Further research exploiting the longitudinal nature of *Understanding Society* (and other datasets) is required to test the validity of possible causal pathways.
